# Changes in fermentation pattern and quality of Italian ryegrass (*Lolium multiflorum* Lam.) silage by wilting and inoculant treatments

**DOI:** 10.5713/ajas.20.0336

**Published:** 2020-08-21

**Authors:** Chang Liu, Guo Qiang Zhao, Sheng Nan Wei, Hak Jin Kim, Yan Fen Li, Jong Geun Kim

**Affiliations:** 1 Graduate School of International Agricultural Technology, Seoul National University, Pyeongchang 25354, Korea; 2 Research Institute of Eco-friendly Livestock Science, Institute of GreenBio Science Technology, Seoul National University, Pyeongchang 25354, Korea

**Keywords:** Fermentation, Inoculant, Italian Ryegrass, Silage, Wilting

## Abstract

**Objective:**

This study was conducted to investigate the effects of wilting and microbial inoculant treatment on the fermentation pattern and quality of Italian ryegrass silage.

**Methods:**

Italian ryegrass was harvested at heading stage and ensiled into vinyl bags (20 cm×30 cm) for 60d. Italian ryegrass was ensiled with 4 treatments (NWNA, no-wilting no-additive; NWA, no-wilting with additive; WNA, wilting no-additive; WA, wilting with additive) in 3 replications, wilting time was 5 hours and additives were treated with 10^6^ cfu/g of *Lactobacillus plantarum*. The silages samples were collected at 1, 2, 3, 5, 10, 20, 30, 45, and 60 days after ensiling and analyzed for the ensiling quality and characteristics of fermentation patterns.

**Results:**

Wilting treatment resulted in lower crude protein and *in vitro* dry matter digestibility and there were no significant differences in acid detergent fiber (ADF), total digestible nutrient (TDN), water-soluble carbohydrate (WSC), ammonia content, and pH (p>0.05). However, wilting treatment resulted in higher ADF and neutral detergent fiber content of Italian ryegrass silage (p<0.05), and the WNA treatment showed the lowest TDN and *in vitro* dry matter digestibility. The pH of the silage was higher in the wilting group (WNA and WA) and lower in the additive treatment group. Meanwhile, the decrease in pH occurred sharply between the 3–5th day of storage. The ammonia nitrogen content was significantly lower in the additive treatment (p<0.05), and wilting had no effect. As fermentation progressed, the lactic and acetic acid contents were increased and showed the highest content at 30 days of storage.

**Conclusion:**

The wilting treatment did not significantly improve the silage fermentation, but the inoculant treatment improved the fermentation patterns and quality of the silage. So, inoculation before ensiling is recommended when preparing high quality of Italian ryegrass silage, and when wilting, it is recommended to combine inoculation for making high quality silage.

## INTRODUCTION

Two types of ryegrass, perennial and annual, are being cultivated in worldwide. Italian ryegrass (IRG, *Lolium multiflorum* Lam.) is an herbaceous annual or biennial grass that is grown for silage, and as a cover crop. In the United States, *Lolium multiflorum* is sometimes used as a winter cover crop to prevent erosion, build soil structure, and suppress weeds. It grows on about 1 million ha in the humid, southern United States, being used primarily for winter pasture in clear seeding and in dormant bermudagrass sods [1].

Korea has four distinct seasons, with crop cultivation during winter being extremely limited. Also, cultivation using paddy fields is dominant because the forage production bases are weak [2]. IRG is one of the wintering crops that is grown after rice cultivation. About 135 thousand ha of IRG were cultivated in 2015 and being approximately 52% of total forage cultivation areas [3]. IRG is one of the fastest growing grasses available to farmers. It is widely distributed throughout temperate and tropical or subtropical regions of the world and is one of China’s major forage crop used either fresh green-chop, hay, or silage [4].

Unfortunately, rains come often at the proper harvest time of the Italian ryegrass in Korea (early May to mid May), which restrict the storage methods. Most farmers stored IRG in the form of silage, and some farmers are trying to store as hay. Produced silage (round bale) is wrapped in plastic vinyl and distributed throughout the country [2]. However, due to the lack of silage preparation technology, the quality of the sold silage is uneven and there is a distrust between producers and consumers.

Ensiling forage crop is well known method of conservation for a shortage season. Lactic acid bacteria (LAB) convert water soluble carbohydrates (WSC) under anaerobic conditions into lactic acid. As a result of the pH decline, the silage is well preserved. Acidification of well fermented silage inhibits undesirable microorganisms. During the fermentation process, competition takes place between LAB and undesirable microorganism, and fermentation quality always depend on the result of the competition [5].

Dry matter (DM) content of raw materials has a great influence on silage fermentation, which affects all fermentation characteristics, pH level and quality parameter of silage. Ensiling with low DM content around 25% could cause inferior fermentation and high pH level deducing serious DM loss, compared with higher DM content. At less than 300 g/DM kg may also generate an increase in seepage loss and expedite *clostridial* fermentation reducing voluntary intake [6]. But wilting reduces the amount of fermentable carbohydrate required to properly preserve the silage and restricts the growth of undesirable microorganism.

The application of silage additives is normally recommended to ensure and improve silage fermentation. At present, LAB inoculants are the main additives in many parts of the world [7]. LAB additives usually increase the rate of lactic acid production, thereby accelerating the pH decline and reducing post-harvest proteolysis. In addition, rapid acidification results in the inhibition of detrimental microorganisms [8].

Generally, wilting results in lower WSC content, extensive protein breakdown and sometime higher total volatile fatty acid (VFA) during ensiling. Wilting also affects the chemical composition, DM losses, silage fermentation and animal performance [9].

This study evaluated the effect of wilting and inoculant on fermentation dynamics and qualities of IRG silage.

## MATERIALS AND METHODS

The experiment was split-plot design with three replications. The main plot was four treatments (NWNA, no-wilting no-additive; NWA, no-wilting with additive; WNA, wilting no-additive; and WA, wiling with additive) and sub-plot was silage opening dates (1, 2, 3, 5, 10, 20, 30, 45, and 60).

### Silage preparation

Italian ryegrass (IRG) was cultivated in an experimental field of Pyeongchang campus, Seoul National University (37.32°N, 128.26°E, 550 m ASL). “Kogreen” variety, developed by the National Institute of Animal Science, was seeded on about 1 ha area on September 27, 2016 at a seeding rate of 40 kg/ha. At seeding date, 40 kg/ha of nitrogen, 150 kg/ha of phosphate, and 75 kg/ha of potassium were applied as fertilizer. An additional 100 kg/ha of nitrogen and 75 kg/ha of potassium fertilizer were applied in early March 2017.

IRG was harvested at 16 May 2017 using mower conditioner (Novacat 301, Pöttinger, Harvest width 3.04) and chopped into about 2 to 3 cm pieces using a forage cutter (Richi Machinery Co., Ltd, Henan, China). The ryegrass harvested for wilting treatment was dried in the field for 5 hours. After manual mixing, chopped IRG was treated with silage inoculant (“Chungmi-Lacto”, Chung-mi Co., *Lactobacillus plantarum*). Recommended level (10^6^ cfu/g fresh matter) of inoculant was dissolved in tap water (1 L per 1 g of inoculant) and sprayed (Air spray gun, Newstar Co., China) into mixed samples. Thereafter, approximately 600 g treated material was packed into vinyl bag (28 cm×36 cm, Korea), air was taken out, sealed (vacuum sealer, Zhejiang Hongzhan Packing Machinery Co., Ltd, China) and stored at the ambient temperature (22°C to 28°C) in the shade.

### Laboratory analysis

Three vinyl bag silos per treatment were randomly opened on 1, 2, 3, 5, 10, 20, 30, 45, and 60 days after ensiling, respectively. Two subsamples per vinyl bag silo were retained for further analysis. One subsample (about 300 g) was dried at 65°C in a forced-air drying oven for 72 h and then used to determine DM content and other chemical compositions, including acid detergent fiber (ADF), neutral detergent fiber (NDF), crude protein (CP), *in vitro* dry matter digestibility (IVDMD), and WSC. Another subsample (about 300 g) was stored at −80°C in a refrigerator and subsequently used for sequential determination of silage acidity (pH), organic acids, microorganisms, and ammonia nitrogen.

Dried samples were ground to pass through a 1-mm screen and kept in double-plug type plastic bottles for analysis. The CP was determined using the Kjeldahl method [10]; ADF and NDF were measured following the method of Goering and Van Soest [11] using an Ankom200 Fiber Analyzer (Ankom Technology, Macedon, NY, USA). We determined the IVDMD of the IRG silage using the two-stage technique described by Tilley and Terry [12] for a period of 72 h using Ankom II Daisy Incubators (Ankom Technology, USA). The total digestible nutrient (TDN) content was estimated as TDN (%) = 88.9–(0.79×ADF) following the method of Holland et al [13].

A frozen 10 g sample of each silage (three replications per treatment) was macerated with 90 mL of distilled water for 30 min. in a shaker and filtered, and the filtrates were used to measure pH with a pH meter (HI 9024; Hanna Instruments Ltd., Leighton Buzzard, UK). 10 g sample of each silage was macerated with 90 mL of distilled water for 24 h and filtered through filter paper (#6). The filtrates were analyzed for VFA and lactic acid contents. The VFAs were analyzed by Kim et al [14] using gas chromatography (Model 3400; Varian Co., Harbor City, CA, USA), and lactic acid was analyzed by high performance liquid chromatography (HP-1100; Hewlett-Packard Co., Palo Alto, CA, USA). WSC was determined using the anthrone method of Thomas [15] and NH_3_-N concentrations were analyzed by the method of Chaney and Marbach [16] using a spectrophotometer (UVIDEC-610; Jasco Co., Tokyo, Japan).

### Statistical analysis

Data on fermentation dynamics and chemical composition were subjected to two-way analysis of variance with the fixed effects of treatments, ensiling days and interaction (treatment ×ensiling days) using the general linear model procedure of SAS ver 9.1 (SAS Institute, Cary, NC, USA) [17]. Least significant difference tests were used to determine specific differences among means. The level of statistical significance was p<0.05.

## RESULTS

The chemical composition, pH, WSC, and ammonia nitrogen contents of pre-ensiled IRG are given in Table 1. The CP and IVDMD contents of wilted materials were lower than those of no wilted (p<0.05). The NDF content of NWA treatment was lower than WNA treatment (p<0.05). There was no significant difference in ADF, TDN, WSC, ammonia nitrogen content and pH among treatments (p>0.05).

As presented in Table 2, inoculant increased the CP content of IRG silage, but there was no significant difference (p> 0.05) and wilting increased the NDF content. This result seems to be due to proteolytic degradation of raw materials with wilting. TDN and IVDMD of WNA treated silage was the lowest among treatments (p<0.05).

Table 3 presents the results for silage fermentation characteristics. The DM content of wilted silages were significantly higher than those of no-wilted (p<0.05), but inoculation had no effect on DM. The pH of silage was significantly lowered by inoculation and the pH of wilted silage was higher (4.12 and 4.66). Inoculation increased the lactic acid content of both wilted and non-wilted silages but, wilting lowered the lactic acid content of IRG silage. Acetic and butyric acid contents were lower in the silages treated with both additive and wilting and the lowest in NWA silage. The residual contents of WSC after the silage fermentation were significantly higher (60.4 and 57.8 g/kg) in the additive treatment (p<0.05). The ammonia nitrogen content was significantly higher in the non-inoculated silage (p<0.05).

Table 4 shows the change in forage quality from 1 to 45 days after silage preservation. The CP content was increased in NWA and WA treated silages during fermentation periods and there was not a significant difference between inoculated silages (NWNA and WNA silage). But CP content was slightly increased in inoculated silage during fermentation. There was significant difference among treatment and number of fermentation days. NWNA silages were significantly higher in each elapsed day and lower in WNA and WA silage. In addition, CP in IVDMD was not different at the early stage of fermentation but decreased after 30 days. However, there was no significant difference in NWNA treatment. The ADF and NDF contents tended to increase with fermentation and wilted silages were higher. However, there was no significant difference in the ADF and NDF content among fermentation periods of NWNA silage (p>0.05). On the other hand, the TDN content, estimated as ADF content, of NWNA silage showed no significant difference according to the fermentation period.

**Figure 1:**
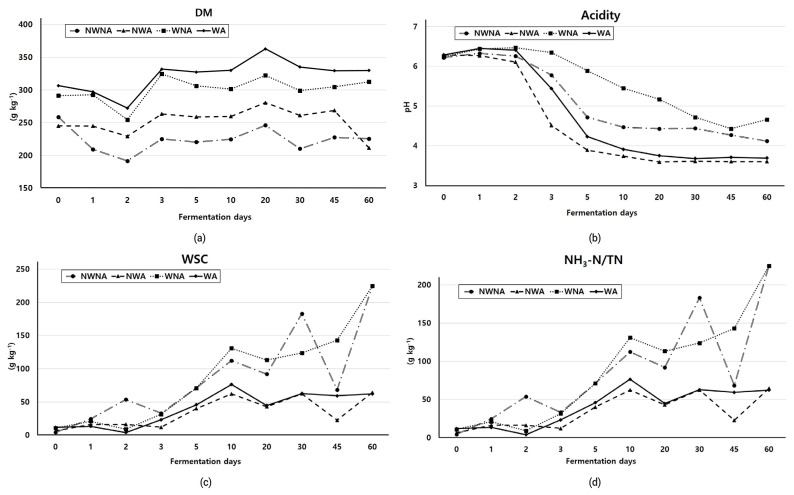
Effect of wilting and inoculant on dry matter (DM, a), acidity (pH, b), water soluble carbohydrate (WSC, c) and ammonia nitrogen/total nitrogen (NH_3_-N/TN, d) content of Italian ryegrass silage. ^1)^ NWNA, no-wilting no-additive; NWA, no-wilting with additive; WNA, wilting no-additive; WA, wilting with additive.

Figure 1 shows the DM content, pH, WSC and ammonia nitrogen content of the silage. The DM content increased continuously as fermentation proceeded but decreased slightly after 20 days. Wilted silages showed the higher DM content among treatments. The pH of silages was significantly decreased from 3 to 5 days after fermentation, and the inoculated silages were significantly lower (p<0.05). In addition, wilting treatment generally delayed the pH decrement. WSC content increased until the 2nd day of storage and then decreased sharply again, while WNA silage was highest at 3 days and decreased. Overall, the WSC content of 45th days’ silage was significantly lower in no inoculated silage (p<0.05). Change in ammonia nitrogen content was significantly lower in the additive treated silage (p<0.05) and tended to increase during the fermentation periods. In particular, the silage treated with additive remained near 50 g/kg, but the silage without additive continued to increase.

Table 5 shows the changes of organic acid content during silage fermentation. Lactic acid content increased with fermentation and decreased at 30 days after conservation at the highest level in all treatments. In addition, lactic acid content was significantly increased by inoculant treatment and highest in NWA silage (p<0.05). Acetic acid content increased for all treatments and then decreased after day 30 but for WA it kept on increasing. Butyric acid content was not detected in the inoculant-treated silage or slightly differentiated by 45 days. In NWNA silage, the butyric acid content was detected after 10 days and the content was continuously increased. On the other hand, the lactic/acetic acid content ratio was significantly higher in the additive treated silage, indicating that homo type fermentation was dominant.

## DISCUSSION

### Analysis of fresh Italian ryegrass

Wilting has a big impact on the fermentation pattern of silage. In this experiment, wilting decreased the CP, NDF, and IVDMD content of raw materials. Kim et al [2] reported that IVDMD decreased with increasing wilting period in rye silage, but increased fiber content. Fitzgerald [18] observed that wilting decreased CP content, but Cottyn et al [19] reported that there was no significant difference.

On the other hand, the WSC content of the material was not significantly different among treatments, and the average level was 153.4 g/kg. Parker [20] stated that the minimum WSC content required for silage fermentation should be 25 to 30 g/kg and the wilted silage should be higher than 38 g/kg. In this experiment, the WSC levels were high. McDonald et al [6] observed that the WSC content varies depending on the species, growth, daily time, light intensity, temperature, and fertilization level and reported that the range recorded in the major five grasses was between 5 to 315 g/kg.

### Forage quality of Italian ryegrass silage

After 60 days of fermentation, the CP content of IRG silage was low in the wilting group, and the inoculant treatment increased CP content. This was judged to be the result of lowering protein degradation by improving fermentation pattern. Kennedy [21] also reported that CP content was significantly higher in the LAB treatment.

On the other hand, ADF and NDF contents were higher and IVDMD contents were lower in WNA treatment silage. The wilting treatment increased the NDF content of the raw material, which is believed to have increased since the high level was maintained even after fermentation.

Comparing the IVDMD before and after silage preparation, the IVDMD of silage was lowered (763.4 vs 735.0 g/kg). Kim et al [2] also showed that the IVDMD of rye silage was lowered by the silage fermentation, which is consistent with this test. Wilkins [22] reported that digestibility tended to decrease by wilting.

After 60 days of storage, the DM content of IRG silage tended to increase in wilted silage but the addition of the inoculant had no significant effect on DM. Keady and Murphy [23] also reported that LAB additives did not affect the dry matter content of silage.

### Silage quality analysis

The most important changes in silage quality was in pH. Wilting resulted in higher silage pH and inoculant was lower. Many studies have reported that LAB treatment lowers the final pH of silage. Wilting increased final pH of silage by increasing the DM content of the raw material. Wilting has negative effect on silage acidity due to increasing DM content and restricted fermentation [24].

The inoculant treatment increased the lactic acid content of the silage, but the wilting treatment resulted in a decrease. This is the result of limited fermentation due to the decrease in moisture content. Kim et al [2] also found that lactic acid content decreased with prolonged wilting period in rye silage. On the other hand, acetic acid and butyric acid contents were decreased by treatment with inoculant. In general, it was reported that LAB additives increased lactic acid and decreased butyric acid contents [25].

The NH_3_-N/total nitrogen (TN) ratio, which indicates the degree of proteolytic degradation, was reduced by LAB treatment, and according to Haigh [25], when the NH_3_-N content was less than 10% of the TN, it was classified as high quality silage. So, the silage of this experiment can be classified as good quality. Sharp et al [26] also found that the NH_3_-N content produced by proteolysis is reduced by the treatment of LAB additives. According to the report of Dawson et al [27], wilting grass before ensiling increased silage pH and ammonia nitrogen concentration, results that agree with many previous studies [21]. The concentration of many of the fermentation products in the silage were also reduced because of wilting, indicating a more restricted fermentation in the wilted silage.

### Analysis of fermentation pattern

The contents of CP, IVDMD, ADF, and NDF in IRG silage showed a significant difference with wilting, LAB treatment and number of fermentation days. The LAB treatment did not show any difference in CP content during fermentation, but CP showed a tendency to increase slightly in control (NWNA). IVDMD of silage showed a tendency to decrease with fermentation, but there was no significant difference in NWNA treatments. The contents of ADF and NDF increased with the fermentation progress, but there was no significant difference in NWNA treatment. Keady and Murphy [23] reported that LAB treatment reduced ADF and NDF content of silage but was not significant. But Patterson et al [9] showed a tendency to increase in ADF and NDF content.

During silage fermentation, the pH changes gradually decreased over time and significantly decreased from 3rd day. However, in the WNA treatment, the pH decrease sharply occurred from slightly late 5th day. However, Zhao et al [28] reported that the pH change was stabilized after the sharpest decrease by day 3 in analysis of rice straw silage fermentation pattern.

Wilting increased ammonia nitrogen content. Derbyshire et al [29] and Haigh [25] also reported that ammonia nitrogen of silage increased by wilting. LAB treatment promoted the production of lactic acid in silage and reduced the production of acetic acid and butyric acid. On the other hand, lactic acid/acetic acid ratio showed a tendency to increase by the treatment of LAB additive, which shows that Homo-type fermentation was predominant.

## CONCLUSION

Wilting resulted in lower CP and IVDMD, but no significant differences in ADF, TDN, WSC, ammonia content and pH (p>0.05) of raw materials. However, wilting treatment resulted in higher ADF and NDF content of IRG silage (p<0.05). The pH of the silage was higher in the wilting group (WNA and WA) and lower in the additive treatment group. The decrement in pH occurred sharply on the 3th to 5th day of storage. The ammonia nitrogen content was lower in the additive treatment (p<0.05). As fermentation progressed, the lactic and acetic acid contents increased and showed their highest content at 30 days of storage. In conclusion, the wilting did not significantly improve the silage fermentation, but the inoculant treatment improves the quality of the silage. So, inoculation before ensiling is recommended when preparing high quality of IRG silage, and when wilting, it was recommended to combine inoculation to make high quality silage.

## Figures and Tables

**Table 1:** Effects of wilting and inoculant treatment, prior to ensiling (0 d), on chemical composition of Italian ryegrass

Item	NWNA	NWA	WNA	WA	Mean	LSD (0.05)
DM (g/kg)	258.8^b^	245.3^b^	291.4^a^	306.7^a^	275.5	16.3
CP (g/kg)	99.8^a^	99.2^a^	87.8^b^	90.6a^b^	94.4	9.66
ADF (g/kg)	276.3	275.4	286.6	285.4	280.9	NS
NDF (g/kg)	493.5^ab^	491.2^b^	518.5^a^	515.2^ab^	504.6	26.69
IVDMD (g/kg)	784.6^a^	785.9^a^	742.0^b^	741.0^b^	763.4	32.28
TDN (%)	67.07	67.14	66.26	66.35	66.7	NS
pH	6.22	6.29	6.32	6.29	6.28	NS
WSC (g/kg)	164.1	183.7	153.3	113.0	153.5	NS
NH_3_-N/TN (g/kg)	4.3	6.3	10.8	11.8	8.27	NS

NWNA, no-wilting no-additive; NWA, no-wilting with additive; WNA, wilting no-additive; WA, wilting with additive; LSD, least significant difference; DM, dry matter; CP, crude protein; ADF, acid detergent fiber; NDF, neutral detergent fiber; IVDMD, *in vitro* dry matter digestibility; TDN, total digestible nutrient; WSC, water-soluble carbohydrate; TN, total nitrogen; NS, non-significant.

ab Values with different small letter show significant difference among treatments (p<0.05).

**Table 2:** Effects of wilting and inoculant treatment, after ensiling (60 d), on chemical composition of Italian ryegrass silage

Item	NWNA	NWA	WNA	WA	Mean	LSD (0.05)
CP (g/kg)	97.9^ab^	101.6^a^	88.8^c^	93.3^bc^	95.4	5.71
ADF (g/kg)	298.6^b^	289.8^b^	310.6^a^	297.0^b^	299.0	8.81
NDF (g/kg)	508.2^b^	499.9^b^	540.2^a^	526.4^a^	518.7	15.56
IVDMD (g/kg)	767.1^a^	739.7^a^	698.7^b^	734.6^a^	735.0	34.28
TDN (%)	65.3^a^	66.0^a^	64.4^b^	65.4^a^	65.3	0.69

NWNA, no-wilting no-additive; NWA, no-wilting with additive; WNA, wilting no-additive; WA, wilting with additive; LSD, least significant difference; CP, crude protein; ADF, acid detergent fiber; NDF, neutral detergent fiber; IVDMD, *in vitro* dry matter digestibility; TDN, total digestible nutrient.

a–c Values with different small letter show significant difference among treatments (p<0.05).

**Table 3:** Dry matter (DM) content, pH, organic acid, water soluble carbohydrate (WSC), and ammonia nitrogen content of Italian ryegrass silage in relation to wilting and inoculant treatment (60 d)

Item	NWNA	NWA	WNA	WA	Mean	LSD (0.05)
DM (g/kg)	225.6^b^	211.7^b^	312.5^a^	329.8^a^	269.9	37.4
pH	4.12^b^	3.60^c^	4.66^a^	3.69^c^	4.02	0.12
Lactic acid (g/kg)	23.5^c^	108.1^a^	18.6^c^	74.3^b^	56.1	20.8
Acetic acid (g/kg)	18.8^a^	8.1^c^	14.3^ab^	11.7^bc^	13.2	4.6
Butyric acid (g/kg)	12.8^a^	0	10.5^ab^	0.8^b^	6.0	2.8
WSC (g/kg)	13.0^b^	60.4^a^	15.6^b^	57.8^a^	36.7	14.01
NH_3_-N/TN (g/kg)	224.9^a^	64.4^b^	224.9^a^	62.2^b^	144.1	39.05

NWNA, no-wilting no-additive; NWA, no-wilting with additive; WNA, wilting no-additive; WA, wilting with additive; LSD, least significant difference; DM, dry matter; WSC, water-soluble carbohydrate; NH_3_-N, ammonia nitrogen; TN, total nitrogen.

a–c Values with different small letter show significant difference among treatments (p<0.05).

**Table 4:** Effect of wilting and inoculant on crude protein (CP), acid detergent fiber (ADF), neutral detergent fiber (NDF), *in vitro* dry matter digestibility (IVDMD) and total digestible nutrient (TDN) content of Italian ryegrass silage according to fermentation days

Item	Treatment1)	Ensiling days								Significance2)		

		1	2	3	5	10	20	30	45	T	D	T×D
CP (g/kg)	NWNA	95.0	95.8^B^	95.7	93.4^B^	99.7^A^	97.8^A^	101.6^A^	97.6A^B^	***	***	*
	NWA	97.3^b^	103.2^ab^^A^	96.2^b^	101.4^ab^^A^	101.6^ab^^A^	101.2^ab^^A^	108.6^a^^A^	102.6^a^^A^			
	WNA	85.1	85.4^C^	85.2	89.4^B^	84.3^B^	87.8^B^	88.6^B^	89.8^C^			
	WA	84.0^c^	84.3^bc^^C^	90.8^ab^	88.1abc^B^	89.2abc^B^	89.6abc^B^	89.9abc^B^	94.8^a^^BC^			
IVDMD (g/kg)	NWNA	773.3^AB^	769.2^B^	762.8^B^	773.0^B^	753.8^B^	766.2^A^	781.3^A^	778.5^A^	***	***	***
	NWA	782.4^a^^A^	781.7^a^^A^	786.4^a^^A^	789.9^a^^A^	780.2^a^^A^	768.9^a^^A^	758.4^b^^B^	759.8^b^^B^			
	WNA	734.9^a^^C^	748.5^a^^B^	739.9^a^^B^	742.8^a^^C^	749.6^a^^B^	728.4^ab^^C^	706.0^bc^^C^	697.2^c^^C^			
	WA	746.8^ab^^BC^	753.6^ab^^B^	758.3^a^^B^	756.3^a^^BC^	745.9^ab^^B^	753.7^ab^^AB^	726.4^c^^BC^	736.1^bc^^B^			
ADF (g/kg)	NWNA	280.5^B^	283.7^A^	282.5	281.4	281.7	285.7^AB^	291.2	293.2^B^	***	***	NS
	NWA	271.4^c^^C^	270.2^c^^B^	274.7^bc^	281.3abc	283.9^ab^	274.4^bc^^B^	290.6^a^	286.8^a^^B^			
	WNA	293.3^a^^A^	287.8^c^^A^	290.1^c^	288.0^c^	297.2^bc^	296.7^bc^^A^	306.1^ab^	310.8^a^^A^			
	WA	288.5^b^^AB^	287.8^b^^A^	281.2^c^	288.4^b^	290.6^ab^	289.6^ab^^A^	292.9^ab^	295.9^a^^B^			
NDF (g/kg)	NWNA	500.0^B^	508.5^A^	508.6^A^	492.6^B^	489.3	485.7^BC^	499.1	503.4^C^	***	***	*
	NWA	487.3^ab^^C^	483.7^ab^^B^	479.6^ab^^B^	485.3^ab^^B^	492.1^ab^	472.7^b^^C^	502.1^a^	496.7^a^^C^			
	WNA	523.9^bc^^A^	514.0^cd^^A^	514.3^cd^^A^	508.2^cd^^A^	514.6^cd^	516.1^cd^^A^	532.4^ab^	544.3^a^^A^			
	WA	521.8^a^^A^	520.5^b^^A^	504.4^cd^^AB^	506.1^cd^^A^	507.9^cd^	507.9^cd^^AB^	513.2^bc^	518.6^ab^^B^			
TDN (%)	NWNA	66.74^B^	66.49^B^	66.58	66.67	66.65	66.33^AB^	65.90	65.74^A^	***	***	NS
	NWA	67.46^a^^A^	67.55^a^^A^	67.20^ab^	66.68abc	66.47abc	67.22^ab^^A^	65.95^ac^	66.25^ac^^A^			
	WNA	65.73^a^^C^	66.16^a^^B^	65.98^a^	66.15^a^	65.42^ab^	65.46^ab^^B^	64.72^bc^	64.35^c^^B^			
	WA	66.10^b^^BC^	66.16^b^^B^	66.69^a^	66.11^b^	65.94^bc^	66.02^bc^^B^	65.76^bc^	65.53^c^^A^			

1) NWNA, no-wilting no-additive; NWA, no-wilting with additive; WNA, wilting no-additive; WA, wilting with additive.

2) T, treatment; D, ensiling days; T×D, interaction between treatments and ensiling days; NS, non-significant.

* p<0.05,

*** p<0.001.

a–d Values with different small letter show significant difference among ensiling days in the same treatment (p<0.05).

A–C Values with different capital letter show significant difference among treatments in the same ensiling days (p<0.05).

**Table 5:** Effect of wilting and inoculant on organic acid content and lactic/acetic acid ratio of Italian ryegrass silage according to fermentation days

Item	Treatment1)	Ensiling days								Significance2)		

		1	2	3	5	10	20	30	45	T	D	T×D
Lactic acid (g/kg)	NWNA	ND	ND	ND	8.4^c^^C^	12.1^c^^C^	23.2^b^^C^	60.3^a^^C^	50.8^a^^C^	***	***	**
	NWA	ND	9.7^c^^A^	32.9^c^^A^	80.4^b^^A^	96.2^b^^A^	104.8^b^^A^	136.8^a^^A^	127.4^a^^A^			
	WNA	ND	ND	ND	4.7b^C^	6.9^b^^C^	12.3^b^^C^	42.8^a^^C^	38.4^a^^C^			
	WA	ND	4.5^c^^B^	20.7^b^^B^	54.7^b^^B^	72.6^a^^B^	83.8^a^^B^	103.7^a^^B^	80.6^a^^B^			
Acetic acid (g/kg)	NWNA	ND	ND	ND	22.3^A^	19.4^A^	18.1^B^	23.7^A^	17.2^B^	**	**	**
	NWA	ND	ND	ND	10.7^B^	11.4^AB^	12.8^B^	13.2^B^	11.9^C^			
	WNA	ND	ND	ND	8.4^b^^BC^	9.5^b^^B^	26.0^b^^A^	24.8^a^^A^	21.7^a^^A^			
	WA	ND	ND	ND	7.5^C^	8.5^CB^	8.4^C^	9.7^C^	11.0^C^			
Butyric acid (g/kg)	NWNA	ND	ND	ND	ND	7.4	8.4	10.8^A^	11.3^A^	***	**	**
	NWA	ND	ND	ND	ND	ND	ND	ND	ND			
	WNA	ND	ND	ND	ND	ND	ND	1.4^B^	2.3^B^			
	WA	ND	ND	ND	ND	ND	ND	ND	0.5^B^			
Lactic/ acetic acid	NWNA	0.00	0.00	0.00	0.38^c^^B^	0.62^c^^B^	1.28^b^^B^	2.54^b^^B^	2.95^b^^B^	***	***	***
	NWA	0.00	0.00	0.00	7.51^b^^A^	8.44^b^^A^	8.19^b^^A^	10.36^a^^A^	10.71^a^^A^			
	WNA	0.00	0.00	0.00	0.56^b^^B^	0.73^b^^B^	0.47^b^^B^	1.73^a^^B^	1.77^a^^B^			
	WA	0.00	0.00	0.00	7.29^b^^A^	8.54^b^^A^	9.98^a^^A^	10.69^a^^A^	7.33^b^^A^			

1) NWNA, no-wilting no-additive; NWA, no-wilting with additive; WNA, wilting no-additive; WA, wilting with additive; ND, not detected.

2) T, treatment; D, ensiling days; T×D, interaction between treatments and ensiling days.

** p<0.01,

*** p<0.001.

a–c Values with different small letter show significant difference among ensiling days in the same treatment (p<0.05).

A–C Values with different capital letter show significant difference among treatments in the same ensiling days (p<0.05).
